# The Inhibitory Effect of Curcumin Derivative J147 on Melanogenesis and Melanosome Transport by Facilitating ERK-Mediated MITF Degradation

**DOI:** 10.3389/fphar.2021.783730

**Published:** 2021-11-23

**Authors:** Jinpeng Lv, Ying Yang, Bingyi Jia, Siqi Li, Ximei Zhang, Rongyin Gao

**Affiliations:** ^1^ School of Pharmacy, Changzhou University, Changzhou, China; ^2^ Department of Pharmacy, The First People’s Hospital of Changzhou, The Third Affiliated Hospital of Soochow University, Changzhou, China

**Keywords:** J147, hypopigmentary effects, melanosome transport, ERK pathway, MITF degradation

## Abstract

The therapeutic use of curcumin and chemically modified curcumin (CMC) for suppressing melanogenesis and tyrosinase activity have been recognized. J147 is a modified version of curcumin with superior bioavailability and stability. However, there is no report about the effects of J147 on pigmentation *in vitro* and *in vivo*. In our studies, we investigated the hypopigmentary effects of J147 treatment on melanocytes and explored the underlying mechanism. The present studies suggested that J147 suppressed both basal and α-MSH-induced melanogenesis, as well as decreased melanocyte dendricity extension and melanosome transport. J147 played these roles mainly by activating the extracellular signal-regulated protein kinase (ERK) pathway. Once activated, it resulted in MITF degradation and further down-regulated the expression of tyrosinase, TRP-1, TRP-2, Myosin Va, Rab27a and Cdc42, ultimately inhibited melanin synthesis and melanosome transport. Furthermore, the hypopigmentary effects of J147 were demonstrated *in vivo* in a zebrafish model and UVB-induced hyperpigmentation model in brown guinea pigs. Our findings also suggested that J147 exhibited no cytotoxicity *in vitro* and *in vivo*. Taken together, these data confirmed that J147 may prove quite useful as a safer natural skin-whitening agent.

## Introduction

Skin pigmentation depends on both melanin synthesis and distribution in the epidermis layer. Melanin is produced mainly around the nucleus of melanocytes and stored in melanosomes (Tian et al., 2021). After mature, melanosomes migrated along microtubules and actin filaments to the dendrite tips of the cells and finally to the neighboring keratinocytes to finish the distribution process (Beaumont et al., 2011; Ohbayashi and Fukuda, 2012). Under normal physiological status, melanin protects human skin from ultraviolet (UV) damage, toxic chemicals and other environmental factors (Slominski et al., 2004; Yousaf et al., 2020). However, excessive production and accumulation induces hyperpigmentation and is associated with skin disorders like post-inflammatory melanoderma, melasma and solar lentigines, leading to remarkable psychosocial burden (Pillaiyar et al., 2017). Hence, it is necessary to develop effective and safe skin whitening agents.

In recent years, melanin cell biology has become a broader research field and several important proteins contributing to melanogenesis and melanosome transport have been elucidated, paying the way for identifying melanin synthesis inhibitors. Tyrosinase is exclusively necessary for melanogenesis and inhibition of tyrosinase catalytic action is the most common method to reduce melanin production (D'Mello et al., 2016). Several known tyrosinase inhibitors, including arbutin and kojic acid, have already been developed as cosmetic additives (Ding et al., 2020). KIF5b and Rab27A-Melanophilin-Myosin Va complex contribute to the outward melanosome transport (Ohbayashi and Fukuda, 2012; Noguchi et al., 2014). Cdc42 regulates dendrite elongation, which is essential for melanosome transfer (Luo, 2000). The inhibition of these above proteins could significantly suppress melanosome transport, which is an important mechanism for developing skin whitening agents. Microphthalmia-associated transcription factor (MITF) is a master transcription factor in melanogenesis. Besides, MITF also regulates melanosome transport by inducing the expression of Rab27a and Cdc42 (Noguchi et al., 2016). Multiple signaling pathways participate in pigmentation by regulating the expression level of MITF. Activation of cAMP protein kinase A (PKA) stimulates pigmentation through cAMP response element binding protein (CREB) dependent upregulation of MITF expression (Rodríguez and Setaluri, 2014). Conversely, activation of extracellular signal regulated protein kinase (ERK) inhibits melanogenesis by accelerating MITF degradation (Lv et al., 2020a). Numerous anti-melanogenic agents have been developed which target tyrosinase activity, melanosome transfer, or melanogenic related signaling pathways. However, few inhibitors underwent studies *in vivo* and showed good results (Pillaiyar et al., 2017).

Curcumin is a diarylheptanoid compound isolated from the rhizome of Curcuma longa (Zingiberaceae) and used as a yellow flavor or pigment in foods (Zheng J. et al., 2018). Studies have indicated its various physiological functions, including anti-inflammatory, anti-oxidative, anti-amyloid and anti-tumor activities (Liu et al., 2016; Zheng Y. et al., 2018). Apart from these, curcumin inhibits tyrosinase activity and suppresses melanogenesis in melanocytes (Lee et al., 2010; Tu et al., 2012). But, the poor bioavailability of curcumin limits its application (Karthikeyan et al., 2020). To solve this problem, J147 is developed as a potent compound of curcumin derivative with greater stability and bioavailability (Li et al., 2020). J147 has a neuroprotective effect and is currently in phase I clinical trials for Alzheimer’s disease. However, there is still no report about the effects of J147 on pigmentation *in vitro* and *in vivo*.

In the present work, we aimed to examine whether J147 affect melanin synthesis and melanosome distribution, and further explore the underlying mechanism. Surprisingly, we observed that J147 suppressed both basal and α-MSH-induced melanogenesis, as well as decreased melanocyte dendricity extension and melanosome distribution. J147 played these roles mainly by activating the extracellular signal regulated protein kinase (ERK) pathway. Once activated, it resulted in MITF degradation and further down-regulated the expression of tyrosinase, TRP-1, TRP-2, Myosin Va, Rab27a and Cdc42, ultimately inhibited melanin synthesis and melanosome transport. Finally, the hypopigmentary effects of J147 were demonstrated *in vivo* in zebrafish model and UVB-induced hyperpigmentation model in brown guinea pigs.

## Materials and Methods

### Reagents

J147 (J302241), α-MSH (M118985) and tyrosinase from mushroom (T128536) were obtained from Aladdin (Shanghai, China). We obtained antibodies against Myosin Va (sc-365986), KIF5b (sc-133184), GP100 (sc-393094), Cdc42 (sc-8401), Rab27a (sc-74586), p-JNK (sc-6254), JNK (sc-7345), p-p38 MAPK (sc-166182) and p38 MAPK (sc-398546) from Santa Cruz (CA, USA). The antibodies against MITF (97800), p-MEK (2338), MEK (4694), p-ERK (4370), ERK (4695) were obtained from Cell Signaling Technology (MA, USA). The antibodies against tyrosinase (ab180753), TRP-1 (ab235447), TRP-2 (ab221144), cytokeratin (ab7753) and S100 (ab133519) were obtained from Abcam (Cambridge, UK). p38 inhibitor SB203580 (S1863), ERK inhibitor PD98059 (S1805), BCA protein assay kit (P0012), cell lysis buffer (P0013) and β-actin (AF5001) were obtained from Beyotime (Shanghai, China). RT-qPCR kits (RR036A) were purchased from Takara Biomedical Technology (Beijing, China).

### Cell Culture

B16F10 murine melanocytes were cultured at 37°C and 5% CO_2_ atmosphere in Dulbecco’s modified Eagle’s medium (DMEM) (12100046, GIBCO, USA) supplemented with 10% fetal bovine serum (FBS) (HyClone, USA), 100 U/ml penicillin and 100 μg/ml streptomycin. HaCaT cells were cultured under standard conditions as the same way with B16 cells. Normal human epidermal melanocytes (NHEMs) were obtained from Sciencell Research Laboratories (CA, USA) and cultured in Medium 254 (M254500, GIBCO, USA) supplemented with Human Melanocyte Growth Supplement (S0025, GIBCO, USA). The medium was changed every 2 days.

### MTT Assay

Cell viability was examined by MTT assay (Yun et al., 2020). Briefly, the cells were seeded in 96-well plates and treated with J147 (1–8 μM) for 48 h. Then, the cells were washed with PBS and replaced with MTT solution (20 μL). After incubation for additional 4 h, the supernatant solution was removed and DMSO (200 μL) was added to each well. Finally, the optical absorbance at 570 nm was determined.

### Measurement of Melanin Contents

Cells with a density of 2 × 10^5^ cells/mL were seeded in 6-well culture plates. After 24 h incubation, cells were cultured with different doses of J147 (1, 2, 4 μM) and with or without α-MSH (60 nM) stimulation. After 48 h treatment, cells were harvested and the total melanin in the cell pellet was dissolved in 100 μL of NaOH working solution (1 mol/L, 10% DMSO) at 80°C for 1 h, and the absorbance was measured at 405 nm (Lv et al., 2015; Lv et al., 2019).

Zebrafish embryos were collected and dissolved in lysis buffer. After centrifugation, melanin pigment was dissolved in 500 μL of NaOH working solution (1 mol/L, 10% DMSO) at 80°C for 1 h, and the absorbance was measured at 405 nm.

### Tyrosinase Activity Assay

Cellular tyrosinase activity was examined as described previously (Liao et al., 2017; Lv et al., 2020a). In brief, cells were lysed by cell lysis buffer after washing three times, and then the supernatant for tyrosinase activity assay was obtained by centrifuging the lysates. 100 μL PBS (0.1M, pH 6.5) contenting 10 μg proteins mixed with 100 μL 0.1% L-DOPA. The plate was incubated at 37°C for 1 h, and then optical absorbance at 475 nm was monitored.

The direct effect of J147 on tyrosinase activity was tested by a cell-free system as described previously (Lv et al., 2020a). In brief, the reaction for the determination of mushroom tyrosinase activity was conducted in a 96-well plate and the reaction mixture contained mushroom tyrosinase (10 unit), L-tyrosine (0.03%, 50 μL) and 100 μL PBS (0.1 M, pH 6.5) adding with different concentrations of J147. Following incubation at 37°C for 10 min, absorbance at 475 nm was measured using a microplate spectrophotometer.

### Masson–Fontana Ammoniacal Silver Staining

To detect melanin pigment, skin pieces and melanocytes were fixed in formalin and stained following the standard protocol (Gu et al., 2018; Lv et al., 2020b). In brief, slides were washed 3 times with deionized water and then were incubated in ammoniacal silver solution at room temperature for 12 h. After rinsed well in deionized water, slides were incubated in hypo solution for 5 min. Next, slides were rinsed again and counterstained with neutral red stain for another 5 min. Finally, following thorough rinsing, slides were observed under Nikon-Eclipse-Ti microscope.

### Immunofluorescence for Melanosome Transfer

The coculture system of B16F10 and HaCaT cells were established on confocal dish, as described previously (Lee et al., 2015; Lv et al., 2020c). After J147 treatment, the cells were immunostained with anti-GP100 and anti-Cytokeratin according to the standard protocol. Images were taken from Nikon-Eclipse-Ti microscope.

### Immunohistochemistry for S-100

Immunohistochemistry for S-100 was performed as previously described (Lee et al., 2013; Lv et al., 2020b). A brief description was as follows: slides were blocked with 5% BSA at 25°C for 1 h and then incubated with anti-S-100 primary antibody at 4°C overnight. Next day, slides were washed 3 times with TBST solution and incubated with the secondary antibody. Then, slides were treated with aminoethyl carbazole to develop the sections and were observed under a microscope.

### Reverse Transcription–PCR

Cellular total RNA was extracted by TRIzol reagent and quantified spectrophotometrically. Then, SuperScript II Reverse Transcriptase was used to synthesize single-stranded cDNA following the manufacturing instructions. Oligonucleotide primers were purchased from GenScript (Nanjing, China). The sequences of *MITF* gene primers are 5′-AGA​GCA​GGG​CAG​AGA​GTG​AGT​G -3′, 5′-AAC​TTG​ATT​CCA​GGC​TGA​TGA​TGT​C -3′. The sequences of *GAPDH* gene primers are 5′- AGG​TCG​GTG​TGA​ACG​GAT​TTG-3′, 5′- TGT​AGA​CCA​TGT​AGT​TGA​GGT​CA -3′. PCR products were separated by electrophoresis on 1% agarose gels and detected under ultraviolet light (Lee et al., 2013).

### Western Blotting

Proteins (40 μg) were separated by SDS-PAGE gels and transferred to nitrocellulose filter (NC) membranes by an electrophoretic transfer system (Bio-Rad). NC membranes were blocked with 3% BSA in TBST solution at room temperature for 1.5 h. Then membranes were incubated with primary antibodies at 4°C overnight. Next day, the blots were washed 3 times with TBST solution and then incubated with peroxidase-conjugated secondary antibodies at 25°C for 1 h, and visualized by using enhanced chemiluminescence (Lv et al., 2020d).

### Determination of Melanin Content in Zebrafish Model

Briefly, synchronized embryos were collected and arrayed by pipette (three to four embryos per well with 200 μL embryo medium in 96-well plates). Then, J147 and PTU were dissolved in 0.1% DMSO, and were added to the embryo medium from 35 to 60 h (25 h exposure). The influence of J147 on the melanogenesis of zebrafish was observed under the stereomicroscope (Zheng J. et al., 2018).

### Phenotype-Based Evaluation and UVB-Induced Hyperpigmentation Guinea Pig Model

8 brown guinea pigs (6 weeks, approximately 250–300 g) were purchased from the Institute of Laboratory Animal Science (Beijing, China). These guinea pigs were kept alone in a constant temperature and humidity room under a 12-h light/dark cycle. The separate areas (1 cm diametrical circle) of the back of each animal were exposed to 500 mJ/cm^2^ UVB (Sigma SH-4, Shanghai, China) once a day for 1 week to establish the hyperpigmentation model. Then the vehicle (PEG400/EtOH = 7:3) and J147 (1%) were given to the hyperpigmented areas (20 μL solution per circle) twice a day for 3 weeks. The degree of pigmentation was evaluated by calculating the Δ*L*-value according to the L-value measured by the spectrophotometer (YS3010, 3nh, Shenzhen, China), as follows: Δ*L* = *L* (at each day measured)-*L* (at day 0) (Lee et al., 2013; Lv et al., 2020b). All animal procedures in this study were approved by the animal care and use committee of Changzhou University.

### Statistical Analysis

Each experiment was performed in triplicate and results were averaged. Values were given as mean ± SEM. Statistical analysis among different groups was made using one-way ANOVA, followed by Turkey’s post hoc test for multiple comparison tests. The significant difference was defined when *p* < 0.05.

## Results

### J147 Decreased Melanogenesis and the Number of Dendrites Within B16F10 Cells

**FIGURE 1:**
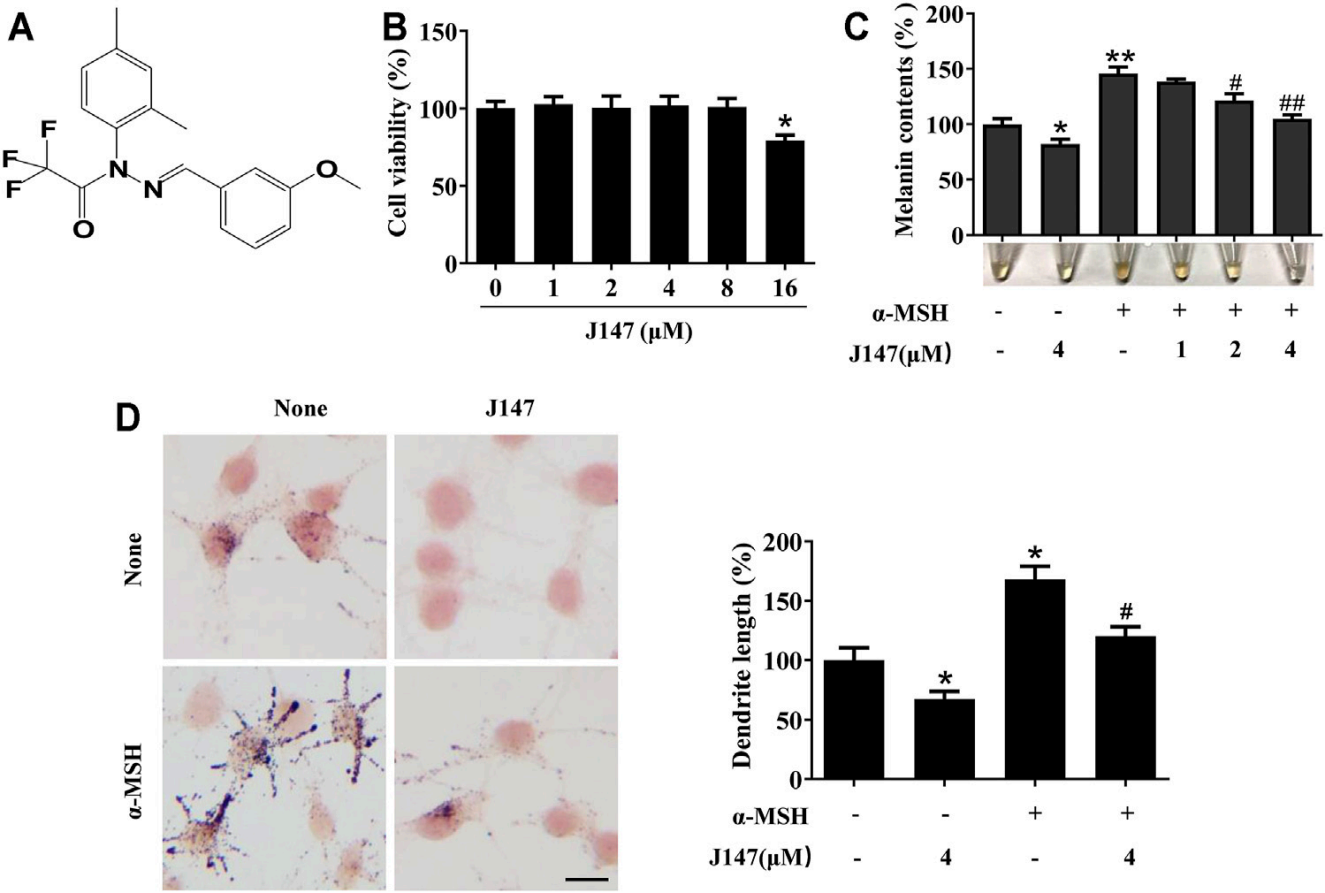
Effect of J147 on melanogenesis in B16F10 cells. **(A)** The chemical structure of J147. **(B)** After incubation of with various concentrations (1–16 μM) of J147 for 48 h, cell viability was determined using MTT assay. **(C)** B16F10 cells were treated with J147 in the presence or absence of α-MSH (60 nM) for 48 h. Melanin contents were measured as described in methods. **(D)** B16F10 cells were treated with J147 for 48 h and were stained with Masson–Fontana ammoniacal silver stain. Bar = 20 μm. Total length of dendrites per cell was measured on the pictures using ruler. Data are expressed as the mean ± SD (*n* = 3). *p < 0.05, **p < 0.01 vs. non-treated cells. ^#^ *p* < 0.05, ^##^ *p* < 0.01 vs. α-MSH-treated cells.

The structure of J147 is shown in Figure 1A. Firstly, a cell viability experiment was performed to investigate if J147 was cytotoxic to B16F10 cells. As shown in Figure 1B, no cytotoxic effects of J147 were observed at a dosing range of 1–8 μM after 48 h. Then, we examined the influence of J147 on melanin synthesis. As shown in Figure 1C, J147 inhibited the basal melanin synthesis. α-MSH significantly promotes melanogenesis in melanocytes. The increase in melanogenesis induced by α-MSH was reversed after J147 treatment. Consistently, Masson–Fontana ammoniacal silver staining showed that J147 remarkably reduced the melanin contents in B16F10 cells with or without α-MSH (Figure 1D). In addition, the number and length of dendrites and the melanin concentration in dendrites were also decreased when compared with untreated cells (Figure 1D). The results suggested that J147 decreased melanogenesis and dendrite formation without cytotoxic effects.

### J147 Inhibited the Cellular Tyrosinase Activity and the Expression of Tyrosinase, TRP-1, TRP-2

**FIGURE 2:**
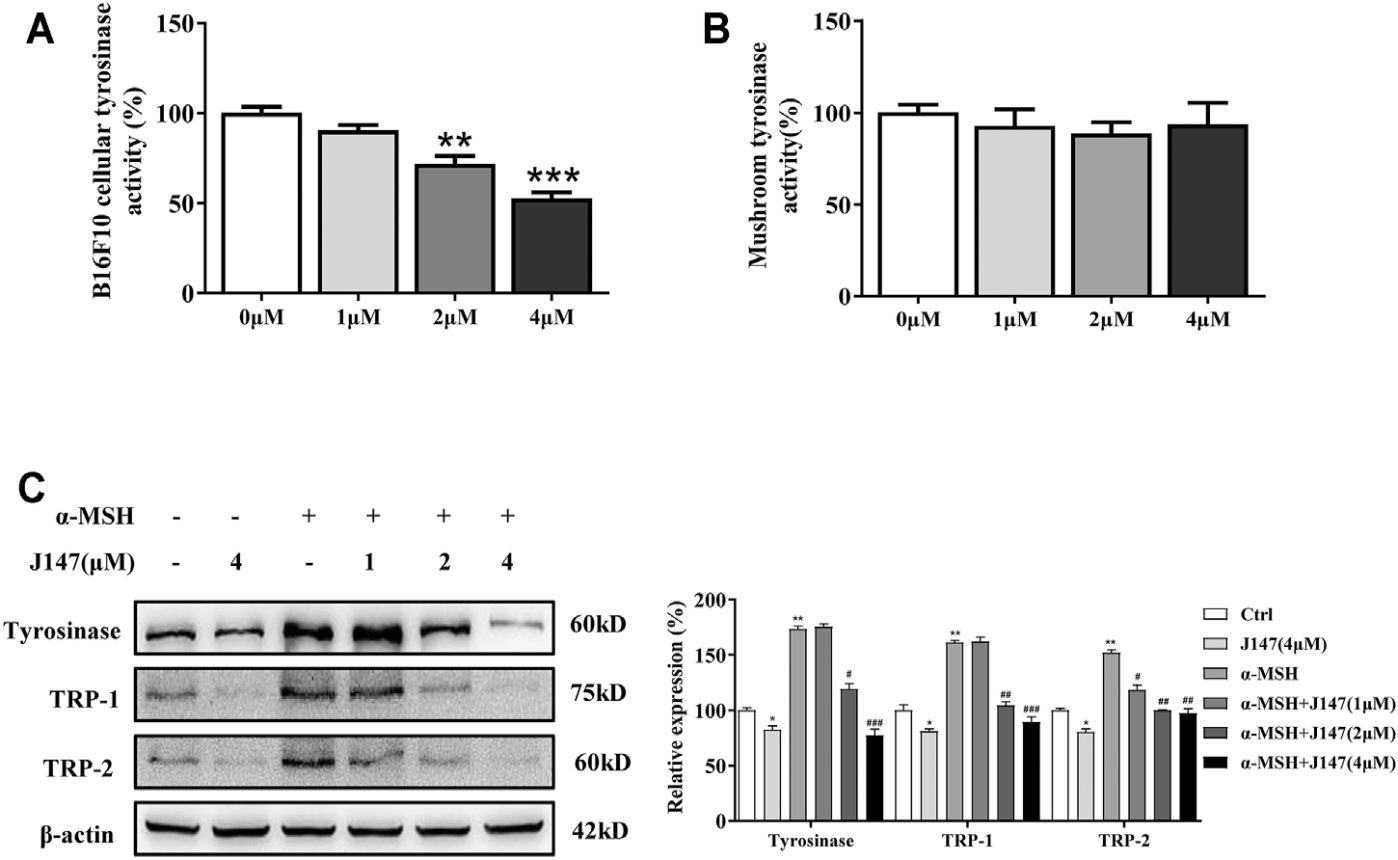
Effect of J147 on the expression of tyrosinase and the activity of tyrosinase. **(A)** Cellular tyrosinase activity was determined by L-DOPA oxization as described in methods. **(B)** Mushroom tyrosinase activity was determined as described in methods. **(C)** B16F10 cells were treated with J147 in the presence or absence of α-MSH (60 nM) for 48 h and western blot was then applied to detect the tyrosinase, TRP-1 and TRP-2 levels. Data are expressed as the mean ± SD (*n* = 3). *p < 0.05, **p < 0.01, ***p < 0.001 vs. non-treated cells. ^#^ *p* < 0.05, ^##^ *p* < 0.01, ^###^ *p* < 0.001 vs. α-MSH-treated cells.

The tyrosinase family of proteins (tyrosinase, TRP-1, and TRP-2) participated in the melanogenesis. Among these, tyrosinases are key enzymes regulating the melanin biosynthetic pathway (D'Mello et al., 2016). To determine whether J147 influences tyrosinase activity, we first used L-DOPA oxidation method to determine the effects of J147 on cellular tyrosinase activity. J147 (1–4 μM) was shown to exert the profound inhibitory effects on cellular tyrosinase activity in a dose-dependent manner in B16F10 cells (Figure 2A). Next, mushroom tyrosinase activity assay was performed to determine the direct effects of J147 on tyrosinase activity. As shown in Figure 2B, no inhibitory effects were observed, indicating that J147 did not influence the enzymatic activities of mushroom tyrosinase (Figure 2B). As we know, melanogenesis is controlled by the activity and amounts of those tyrosinases. To investigate if the anti-melanogenic effects of J147 is correlated with the expression of tyrosinase, western-blot analysis was conducted. As shown in Figure 2C, the levels of tyrosinase expression were significantly reduced after J147 treatment with or without α-MSH, and the same results were observed in the TRP-1 and TRP-2 expression. These observations indicated that J147 inhibited cellular tyrosinase activity and melanogenesis by reducing the expression levels of tyrosinase, TRP-1, TRP-2.

### J147 Inhibited Melanosome Transport by Regulating the Expression of Myosin Va, Rab27a and Cdc42

**FIGURE 3:**
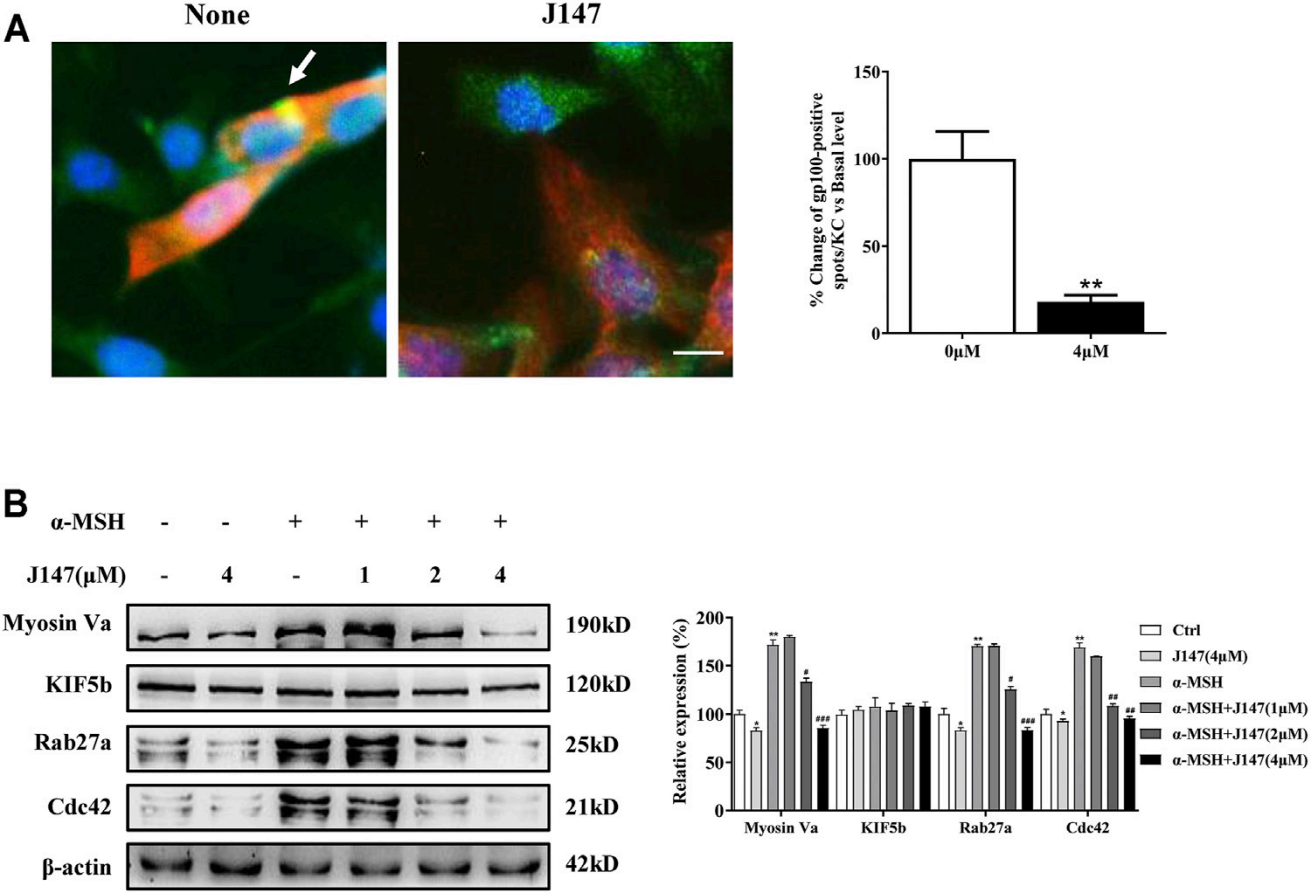
Effect of J147 on melanosome transport in the B16F10 and HaCaT cells co-culture model. **(A)** In cocultured B16F10 and HaCaT cells, melanosome with yellow signal (arrow) were evident in cytokeratin-positive HaCaT cells. Melanosome labeling with GP100 (green). HaCaT cells labeling with cytokeratin (red). Bar = 20 μm. Quantification of melanosomes transferred to HaCaT cells. 20 cells/condition were assessed in each of three independent experiments. **(B)** B16F10 cells were treated with J147 in the presence or absence of α-MSH (60 nM) for 48 h and western blot was then applied to detect the Myosin Va, KIF5b, Rab27a and Cdc42 levels. Data are expressed as the mean ± SEM (*n* = 3). *p < 0.05, **p < 0.01 vs. non-treated cells. ^#^ *p* < 0.05, ^##^ *p* < 0.01, ^###^ *p* < 0.001 vs. α-MSH-treated cells.

Human skin pigmentation is determined by the melanin synthesis as well as the distribution of melanin. In mammalian melanocytes, melanin is mainly made in the cell body and migrates along actin filaments and microtubules to dendrites, and finally to the neighboring keratinocytes to finish the distribution process (Ohbayashi and Fukuda, 2012). As is shown in Figure 1D, J147 markedly decreased the dendrite formation. Furthermore, the melanin concentration in dendrites was also reduced as the melanin pigment was aggregated in the perinuclear regions. Next, we co-cultured B16F10 and HaCaT cells to investigate if J147 influences melanosome transfer to keratinocytes by confocal microscopy. The distribution of melanin was clearly seen in the HaCaT cells in the co-culture model (Figure 3A). In contrast, while the co-culture model was treated with J147 for 48 h, melanosome granular signals in HaCaT cells were significantly reduced (Figure 3A).

To further clarify the underlying mechanism of the inhibition effects of melanosome transfer by J147, we examined several crucial factors involved in melanosome transport. KIF5b mediates outward melanosome transport along microtubules, and the Rab27a-Melanophilin-Myosin Va complex contributes to melanosome transport along actin filaments in melanocytes (Reiner et al., 2020). In addition, Cdc42 stimulates dendrites formation in melanocytes, which also plays an essential role in melanosome transport. As shown in Figure 3B, the expression of Cdc42, Myosin Va and Rab27a, but not KIF5b was significantly decreased after J147 treatment in the condition of with or without α-MSH. Our findings indicated that J147 inhibited melanosome transport by decreasing the expression of Myosin Va, Rab27a and Cdc42.

### J147 Accelerated MITF Degradation

**FIGURE 4:**
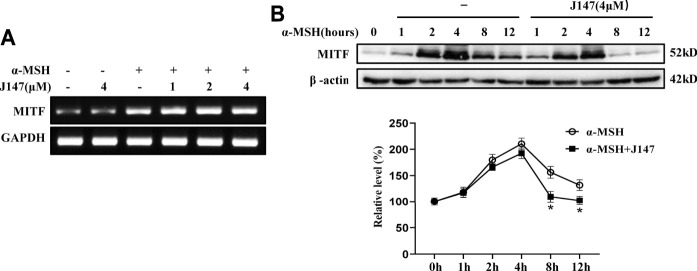
Effect of J147 on the expression of MITF in B16F10 cells. **(A)** B16F10 cells were treated with α-MSH (60 nM) in the presence or absence of J147 for 4 h and RT-PCR was then applied to detect MITF gene expression. **(B)** B16F10 cells were treated with α-MSH (60 nM) in the presence or absence of J147 for different times and western blot was then applied to detect MITF protein levels. Data are expressed as the mean ± SD (*n* = 3). *p < 0.05 vs. α-MSH-treated time-matched control group.

MITF is one of the key regulators for melanogenesis and it controls the gene transcription of tyrosinase, TRP-1, TRP-2, Cdc42 and Rab27a (Noguchi et al., 2016; Kim et al., 2019). We first examined the influence of J147 on MITF transcripts. As shown in Figure 4A, α-MSH remarkably increased MITF transcription, however, no change was observed after J147 treatment, indicating that J147 does not downregulate MITF expression. Next, we examined if J147 influences the translation of MITF. As shown in Figure 4B, after α-MSH treatment, MITF protein levels increased, peaked at 4 h, and began to decline at 8 h (Figure 4B). Differently, the protein levels of MITF did not decrease at 4 h after J147 treatment, but at 8 h the MITF protein levels rapidly declined to undetectable levels, indicating that J147 does not influence the translation of MITF but remarkedly accelerates MITF protein degradation.

### J147 Suppressed Melanogenesis Through the MEK/ERK Signaling Pathway

**FIGURE 5:**
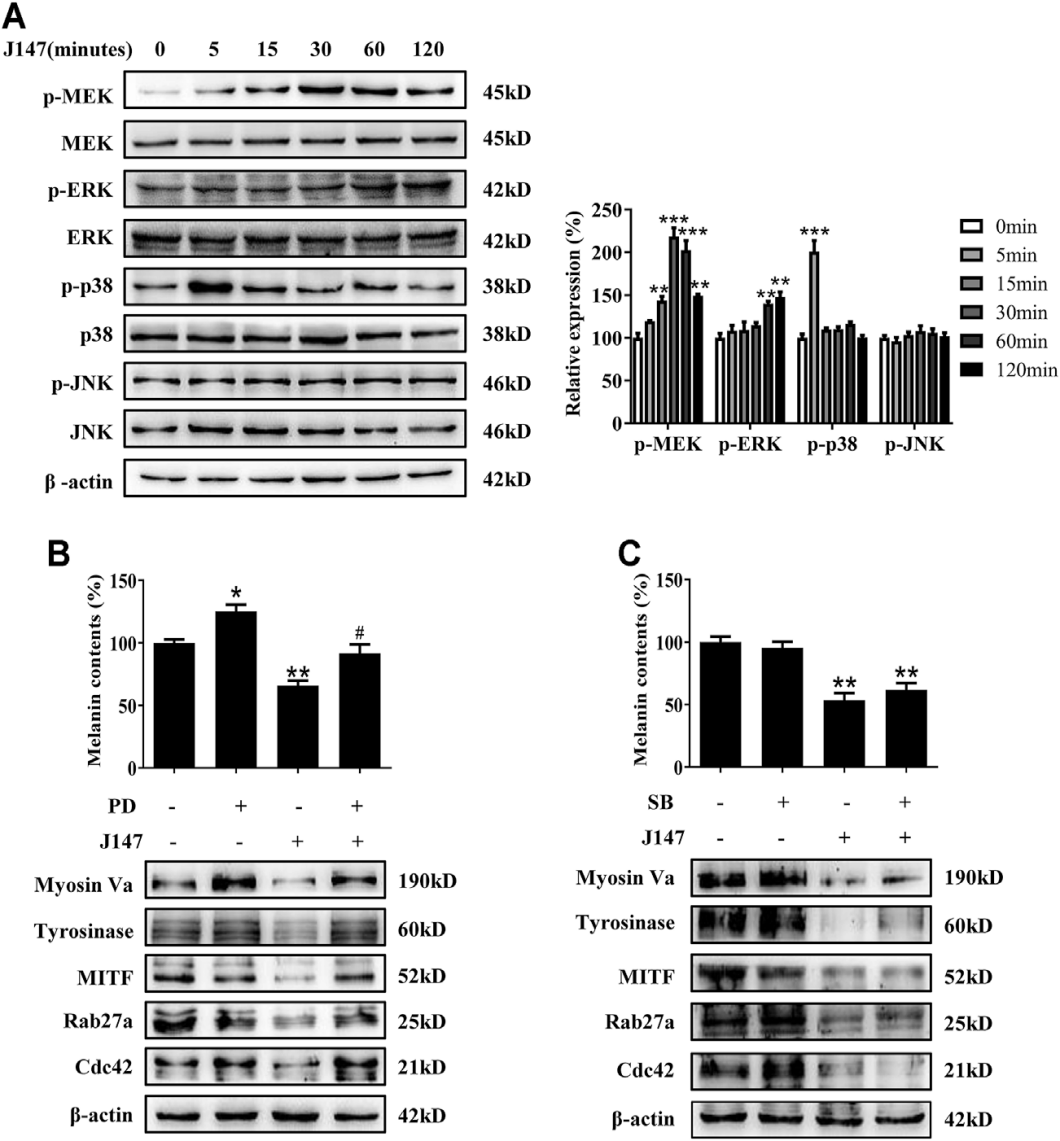
Effect of MAPK pathway on J147-induced hypomelanogenesis. **(A)** B16F10 cells were treated with J147 (4 μM) for the indicated time period (0–120 min), and the phosphorylation of the MEK, ERK, p38 and JNK were measured by western blot. **(B)** B16F10 was pretreated or not with 10 μM SB203580 (the inhibitor of p38) for 1 h before J147 was applied for 48 h. Melanin contents and the expression of Myosin Va, tyrosinase, MITF, Cdc42 and Rab27a were measured as described in methods. **(C)** B16F10 was pretreated or not with 10 μM PD98059 (the inhibitor of ERK) for 1 h before J147 was applied for 48 h. Melanin contents and the expression of Myosin Va, tyrosinase, MITF, Cdc42 and Rab27a were measured as described in methods. Data are expressed as the mean ± SE (*n* = 3). *p < 0.05, **p < 0.01, ***p < 0.001 vs. non-treated cells. ^#^ *p* < 0.05 vs. J147-treated cells.

Mitogen-activated protein kinases (MAPK) signaling pathway, including extracellular signal-regulated protein kinase (ERK), p38 and c-jun N-terminal kinase (JNK), play crucial roles in pigmentation (Lee et al., 2013). ERK phosphorylation is known to facilitate the degradation of MITF and eventually dissipate the melanogenic stimuli, which represents a major negative feedback mechanism (Kwon et al., 2017). However, the function of p38 and JNK pathway remains controversial. Hence, we examined the effect of J147 on the MAPK signaling pathways. As shown in Figure 5A, J147 activated MEK/ERK and p38, whereas no influence was found in the phosphorylation of JNK signaling pathway.

Next, to address if there is an association between J147-induced hypomelanogenesis and ERK and p38 activation. PD98059, a specific inhibitor of the MEK/ERK pathway, suppressed ERK activation by J147 in B16F10 cells (Supplementary Figure S1). SB203580, a specific inhibitor of the p38 pathway, suppressed p38 activation by J147 in B16F10 cells (Supplementary Figure S2). Notably, PD98059 remarkably attenuated the inhibitory effect of J147 on melanin production as well as the protein level of tyrosinase, MITF, Myosin Va, Rab27a and Cdc42 (Figure 5B). However, SB203580 exerted no effects on J147-induced hypopigemntary effects (Figure 5C). Our findings supported that MEK/ERK signaling activation was involved in J147 mediated MITF degradation and hypopigmentary effects.

### J147 Reduced Melanogenesis by Activation MEK/ERK Pathway in Normal Human Epidermal Melanocytes (NHEMs)

**FIGURE 6:**
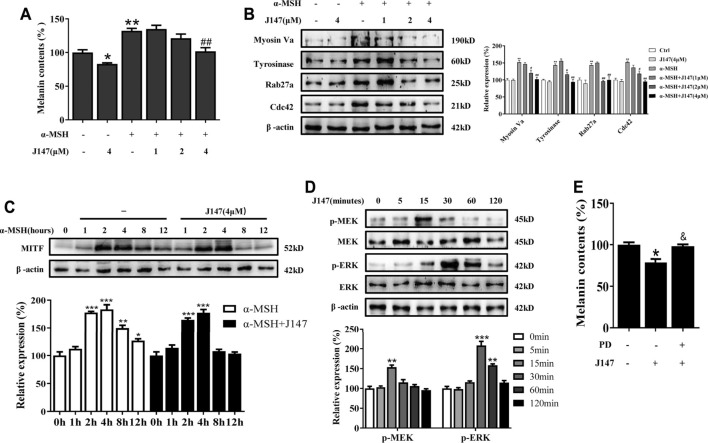
Effect of J147 on melanogenesis in human melanocytes. **(A)** Human melanocytes were treated with J147 for 48 h and melanin contents were measured as described in methods. **(B)** Human melanocytes were treated with J147 for 48 h and the expression of Myosin Va, tyrosinase, Cdc42 and Rab27a were measured using western-blot as described in methods. **(C)** Human melanocytes were treated with α-MSH (60 nM) in the presence or absence of J147 for different times and western blot was then applied to detect MITF protein levels. **(D)** Human melanocytes were treated with J147 (4 μM) for the indicated time period (0–120 min), and the phosphorylation of MEK and ERK were measured by western blot. **(E)** Human melanocytes were pretreated or not with 10 μM PD98059 (the inhibitor of ERK) for 1 h before J147 was applied for 48 h. Melanin contents were measured as described in methods. *p < 0.05, **p < 0.01, ***p < 0.001 vs. non-treated cells. ^#^ *p* < 0.05, ^##^ *p* < 0.01 vs. α-MSH-treated cells. ^&^ *p* < 0.05 vs. J147-treated cells.

We next investigated whether J147 can suppress melanogenesis in normal human epidermal melanocytes (NHEMs). Consistent with the results from B16 melanocytes, J147 also inhibited melanin production in NHEMs (Figure 6A). Western blotting analysis indicated that J147 reduced the protein level of tyrosinase, Myosin Va, Rab27a and Cdc42 in NHEMs (Figure 6B). In addition, degradation of MITF protein was also facilitated after J147 treatment in NHEMs (Figure 6C). MEK/ERK activation was also confirmed to be involved in J147-mediated antimelanogenic in NHEMs (Figures 6D,E).

### J147 Reduced Pigmentation in Zebrafish

**FIGURE 7:**
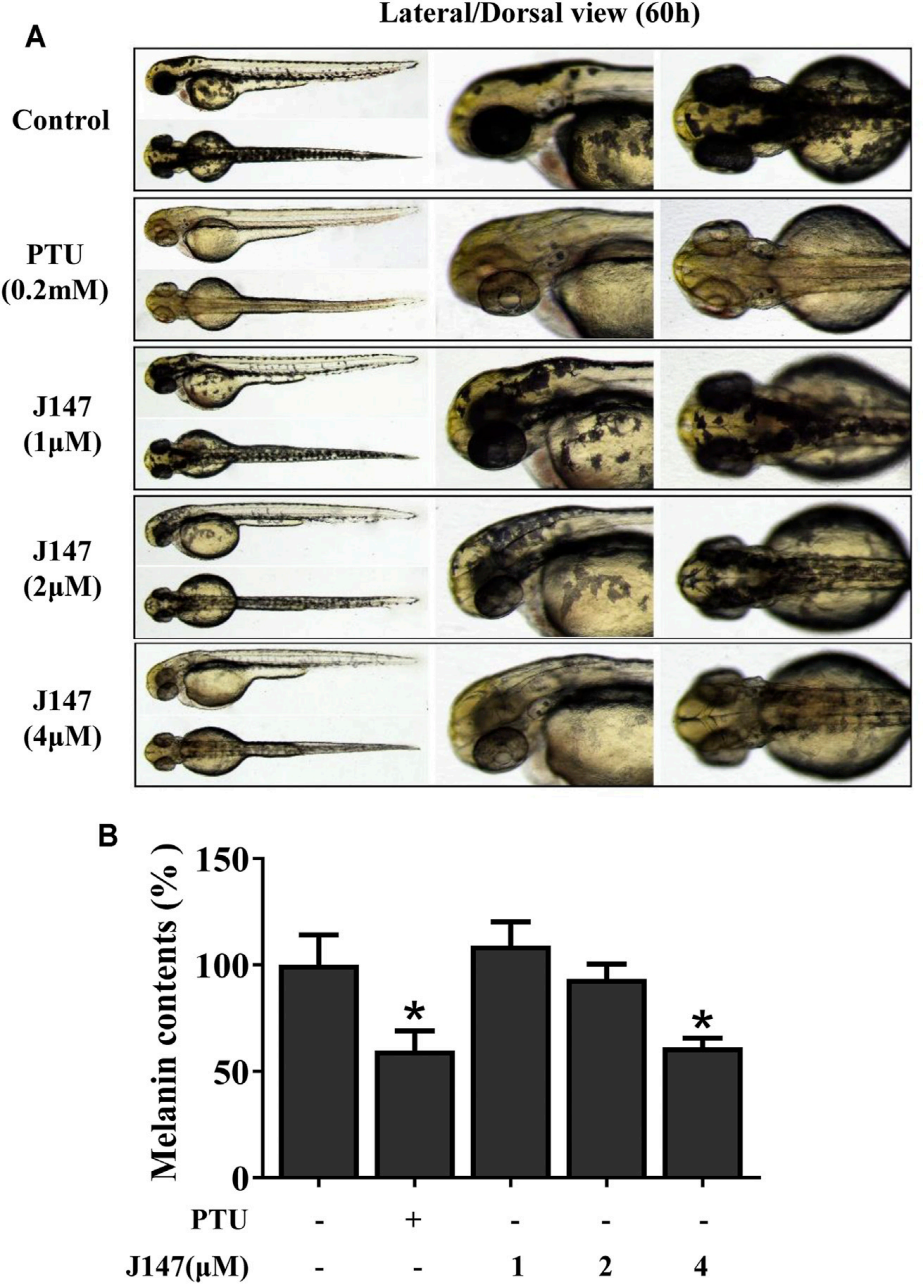
Effect of J147 on pigmentation in zebrafish. **(A)** Representative photographs of zebrafish. Zebrafish embryos were treated with PTU or J147 from 35 to 60 h. The effects on the pigmentation of zebrafish were observed under the stereomicroscope. **(B)** About100 embryos were collected and dissolved in lysis buffer. After centrifugation, melanin pigment was dissolved in 500 μL of NaOH working solution (1 mol/L, 10% DMSO) at 80°C for 1 h, and the absorbance was measured at 405 nm.

Evaluation of candidate compounds based on phenotype provided reliable data for further experiments. The *in vivo* experiment of J147 was conducted in a zebrafish model, as melanin pigments were on the surface of fish, which allows simple observation (Choi et al., 2007). PTU is used widely as a positive control in zebrafish research due to its capacity of inhibiting melanogenesis (Kim et al., 2008). As shown in Figure 7, J147 significantly reduced the body pigmentation, likewise the positive control group treated with PTU.

### J147 Reversed UVB-Induced Hyperpigmentation in Guinea Pig Skin

**FIGURE 8:**
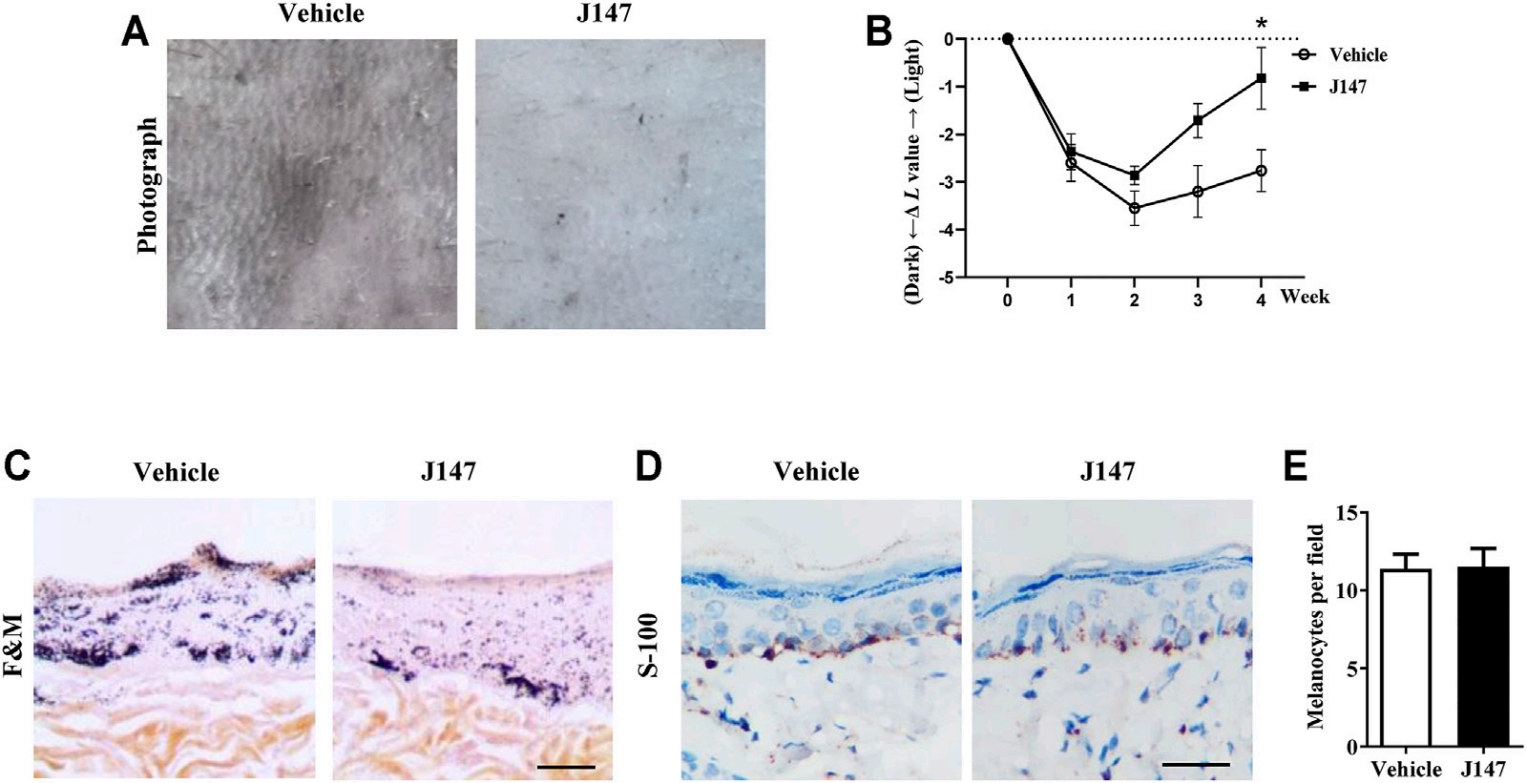
Effect of J147 on pigmentation in guinea-pig skin. **(A)** Representative photographs of dorsal skin of guinea pigs. **(B)** The degree of depigmentation was determined by a chromameter (CR-300; Minolta, Osaka, Japan) once a week for 4 weeks. The Δ*L* value was calculated using the *L* value (brightness index) measured with the chromameter follows: Δ*L* = *L* (at each week measured)—*L* (at day 0). Negative Δ*L* values indicate an UV-induced darkening of the skin. An increase in the Δ*L* value indicates a decrease in hyperpigmentation induced by UV. **(C)** Masson–Fontana ammoniacal silver staining of skin biopsies. **(D)** Immunohistochemical staining of skin biopsies for the detection of S-100 as a melanocyte marker protein. **(E)** Number of melanocytes per microscopic field in skin sections. Bar = 50 μm *p < 0.05 vs. vehicle-treated groups.

We also investigated the anti-melanogenic effects of J147 in UVB-induced hyperpigmentation model in brown guinea pigs. J147 (1%) remarkably inhibited pigmentation when compared with the untreated group as shown by representative photographs of guinea pig skin (Figure 8A). To further evaluate the degree of pigmentation, we used a Spectrophotometer to calculate the *L* value (brightness index). As shown in Figure 8B, the Δ*L* value of the J147 group was markedly higher after 3 weeks treatment compared with the vehicle group, indicating that J147 could reverse hyperpigmentation induced by UVB. Consistent with the above observations, a visible decrease in UVB-induced pigmentation in the epidermal basal layer was observed after J147 treatment by Masson–Fontana ammoniacal silver staining (Figure 8C). In addition, J147 did not affect the melanocyte count as determined by immunohistochemical staining of S100, a melanocyte marker protein (Figures 8D,E). As expected, our findings suggested that J147 has whitening effects on UV-induced hyperpigmentation in guinea pig skin without cytotoxicity.

## Discussion

The Global industry analysts (GIA) have predicted that the global whitening market will reach $31.2 billion by 2024 (Lv et al., 2020b). Many research groups are focusing their efforts to elucidate novel and effective whitening compounds. However, few inhibitors underwent studies *in vivo* and showed good results due to cytotoxicity and weak efficacy (Kim et al., 2008; Pillaiyar et al., 2017). Thus, it is necessary to continue to discover more efficient and safer skin-whitening agents.

The therapeutic use of curcumin and chemically modified curcumin (CMC) for inhibiting tyrosinase activity and melanin formation has been reported recently (Goenka and Simon, 2021). However, the poor bioavailability of curcumin and chemically modified curcumin (CMC) limits its application (Karthikeyan et al., 2020). To solve this problem, J147 is developed as a potent compound of curcumin derivative with greater stability and bioavailability (Li et al., 2020). In this work, we explored the inhibitory influences of J147 on pigment and underlying mechanisms *in vitro* and *in vivo*. Our data showed that J147 not only suppressed the basal melanin production but also attenuated α-MSH-induced melanin increase, without influencing cell viability (Figure 1). It is well known that several skin whitening agents exert their hypomelanogenic effects through directly inhibiting tyrosinase activity. In our studies, J147 did not directly inhibit mushroom tyrosinase activity (Figure 2B), which is inconsistent with previous studies suggesting that curcumin directly inhibited mushroom tyrosinase activity (Lee et al., 2010; Tu et al., 2012). The possible reason is that J147 has no phenolic hydroxyl group, which is a critical functional group for tyrosinase activity inhibition. Western blotting analysis demonstrated that J147 inhibited the expression levels of tyrosinase, TRP-1 and TRP-2 (Figure 2C). Our findings indicated that J147 had whitening effects on melanocytes and acted primarily by downregulating the protein levels of three crucial melanogenic enzymes rather than directly suppressing the tyrosinase activity.

Human skin pigmentation is determined by the melanin synthesis as well as the distribution of melanosomes. The regulation of melanosome transfer from melanocytes to neighboring keratinocytes is an important mechanism for skin whitening. Recently, Goenka et al. reported that chemically modified curcumin analogs suppressed dendricity in melanocytes and inhibited the phagocytosis of FluoSphere beads (melanosome mimics) by HaCaT cells (Goenka and Simon, 2021). The present study expanded upon previous studies and demonstrated that J147 inhibited melanosome transfer to neighboring keratinocytes in the keratinocyte/melanocyte co-culture system (Figure 3A). Mechanisms studies found that the expression levels of Myosin Va, Rab27a and Cdc42 were significantly decreased by J147 treatment, while KIF5b was not affected (Figure 3B). These results suggested that J147 suppressed the melanosome transport along actin filaments and inhibited dendrite extension by downregulating the Myosin Va, Rab27a and Cdc42 expression. However, further investigation of whether J147 affects the melanosome move along microtubules is needed.

MITF is a critical regulator in melanocyte development, differentiation, and survival of melanocytes, and it controls the transcription of genes such as tyrosinase, TRP-1, TRP-2, Cdc42, Rab27a and subsequent promotes melanogenesis and melanosome transport (Noguchi et al., 2016). The present studies showed that J147 had no effect on the expression of MITF genes, suggesting that J147 did not affect MITF transcription (Figure 4A). However, western blotting analysis found that J147 remarkedly accelerated MITF protein degradation (Figure 4B). Numerous studies suggested that MAPK signaling pathway participated in the expression and degradation of MITF (Rodríguez and Setaluri, 2014). Tu et al. reported that curcumin inhibited melanin synthesis through activation of ERK and p38 signaling pathways (Tu et al., 2012). In the present study, J147 increased the phosphorylation of ERK and p38, whereas no outcome was observed in the phosphorylation of JNK. Furthermore, only PD98059, a specific inhibitor of ERK, significantly attenuated the inhibitory effects of J147 on melanin formation and distribution as well as the expression of tyrosinase, MITF, Rab27a, Myosin Va, and Cdc42 (Figure 5). These data indicated that J147 suppressed melanin formation and distribution mainly through facilitating EKR-mediated MITF degradation. Considering that numerous studies have identified the role of p38 MAPK in melanogenic differentiation, the role of J147/p38 signaling pathway in melanocytes needs further study.

Melanogenesis suppressors are very important to the cosmetic industry as skin-lightening agents. Numerous anti-melanogenic agents have been developed, few agents under *in vivo* studies and showed good results (Pillaiyar et al., 2017). J147 is currently in phase I clinical trials for Alzheimer’s disease, which indicates that J147 is a safe and low-toxic agent (Ates et al., 2020). Herein, we investigated the anti-melanogenic effect of J147 *in vivo*. Zebrafish is an extremely advantageous vertebrate model organism due to its gene sequence and organ system are similar to that of humans (Agalou et al., 2018). And melanin pigments are present on the surface of fish, which is simply easy to observe (Choi et al., 2007). As shown in Figure 7, J147 remarkably reduced the body pigmentation in zebrafish, similar to the positive drug PTU. Furthermore, J147 did not affect the developmental growth or survival of zebrafish. UVB-induced hyperpigmentation in brown guinea pig is the other kind of experimental model to investigate the effects of J147 on pigmentation. As shown in Figure 8, obvious whitening effects of J147 were found in which hyperpigmentation caused by UVB exposure after the topical application of J147 for 3 weeks to the dorsal skin of guinea pig. Our results demonstrated that J147 reduced melanin formation in active melanocytes, rather than decreased numbers of melanocytes.

In summary, our study first reported that J147 exerted anti-melanogenic effects *in vitro* and *in vivo*, as well as the underlying mechanism. Specifically, J147 significantly inhibited melanin production, in addition to prevented dendrite extension and melanosome distribution. Mechanistically, J147 played these roles mainly by activating the extracellular signal regulated protein kinase (ERK) pathway. Once activated, it accelerated MITF degradation, subsequently suppressed the protein level of tyrosinase, TRP-1, TRP-2, Myosin Va, Rab27a and Cdc42, and ultimately suppressed melanin production, dendricity extension and melanin distribution. Our results indicated that J147 may be applied as a safer skin-whitening agent without cytotoxicity.

## Data Availability

The original contributions presented in the study are included in the article/Supplementary Material, further inquiries can be directed to the corresponding author.
