# Supplementary material for: The Inhibitory Effect of Curcumin Derivative J147 on Melanogenesis and Melanosome Transport by Facilitating ERK-Mediated MITF Degradation
Source: Front Pharmacol. 2021 Nov 23;12:783730. doi: 10.3389/fphar.2021.783730 (PMC8649847; doi:10.3389/fphar.2021.783730)
Supplement: Supplementary file 1 [file DataSheet1.PDF]

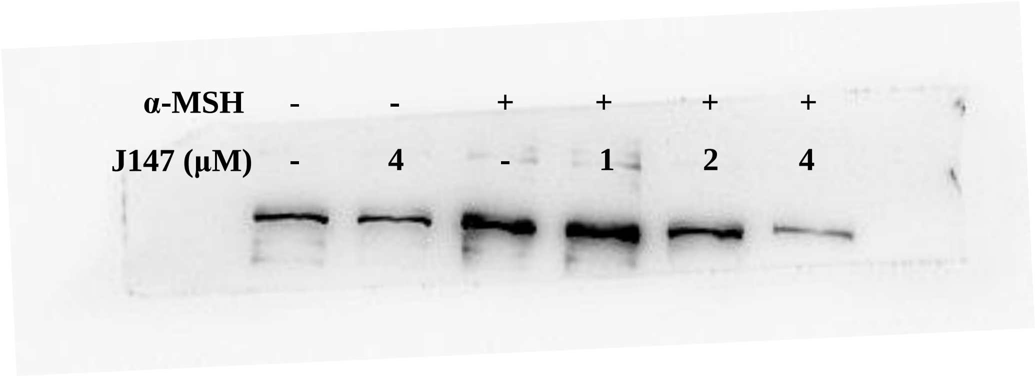

Figure 2C Tyrosinase

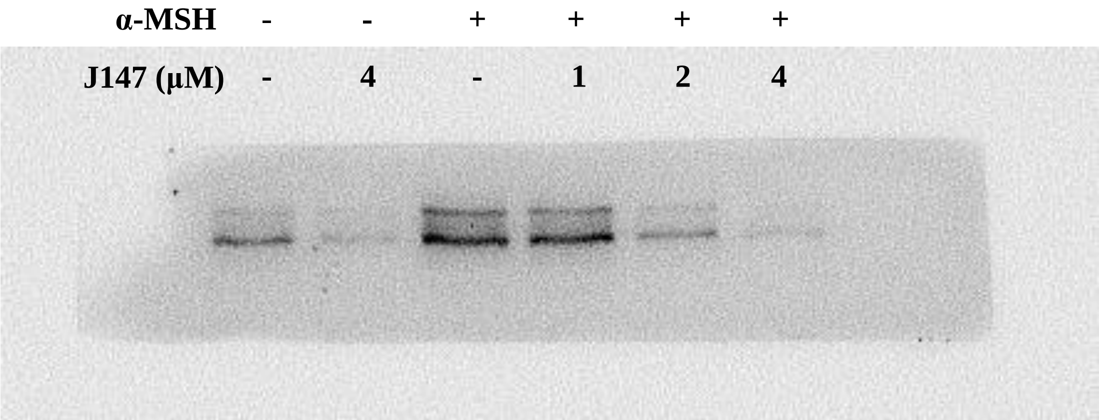

Figure 2C TRP-1

**α-MSH**	**-**	**-**	**+**	**+**	**+**	**+**
**J147 (μM)**	**-**	**4**	**-**	**1**	**2**	**4**

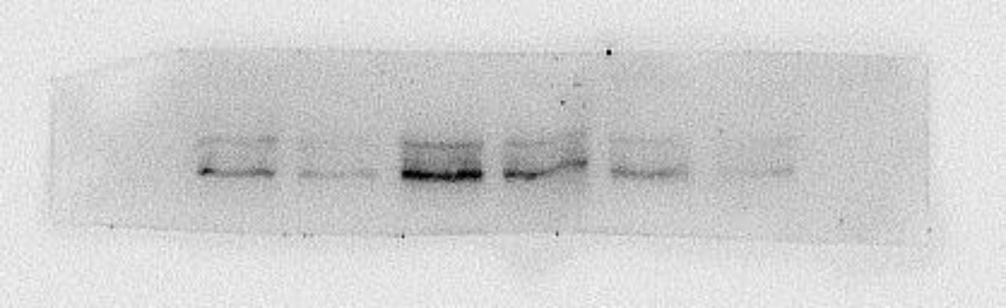

Figure 2C TRP-2

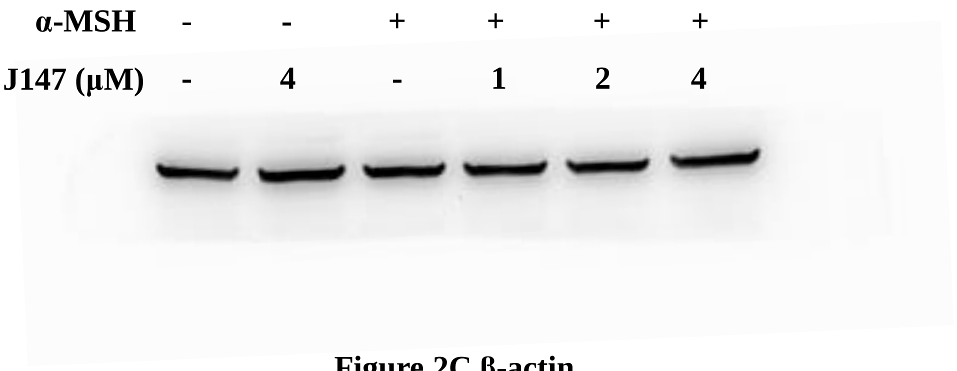

Figure 2C β-actin

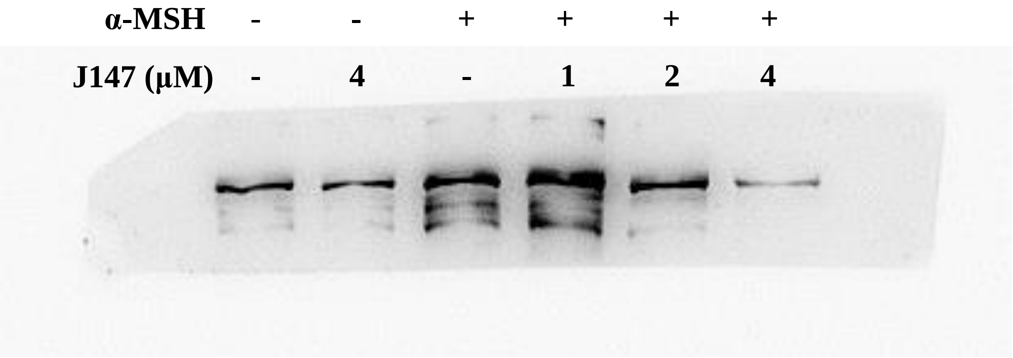

Figure 3B Myosin Va

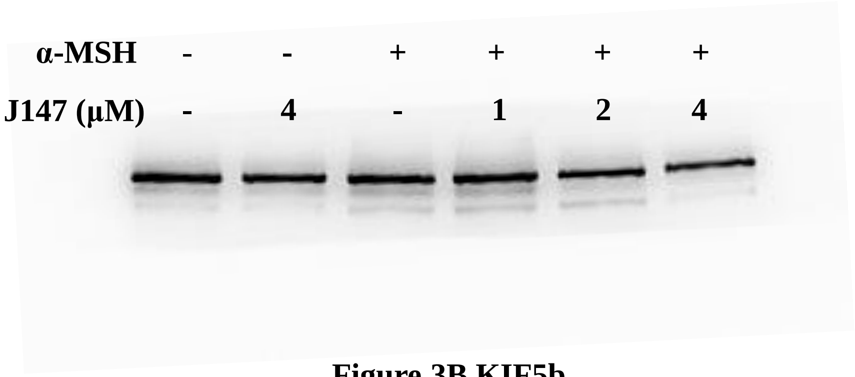

Figure 3B KIF5b

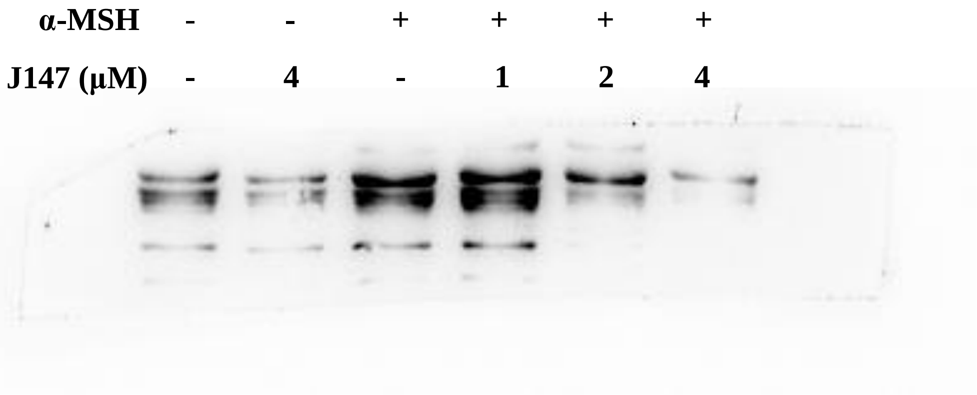

Figure 3B Rab27a

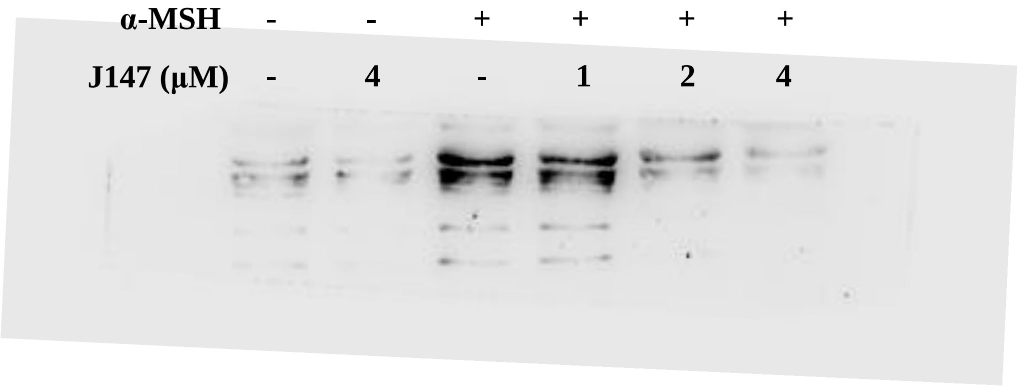

Figure 3B Cdc42

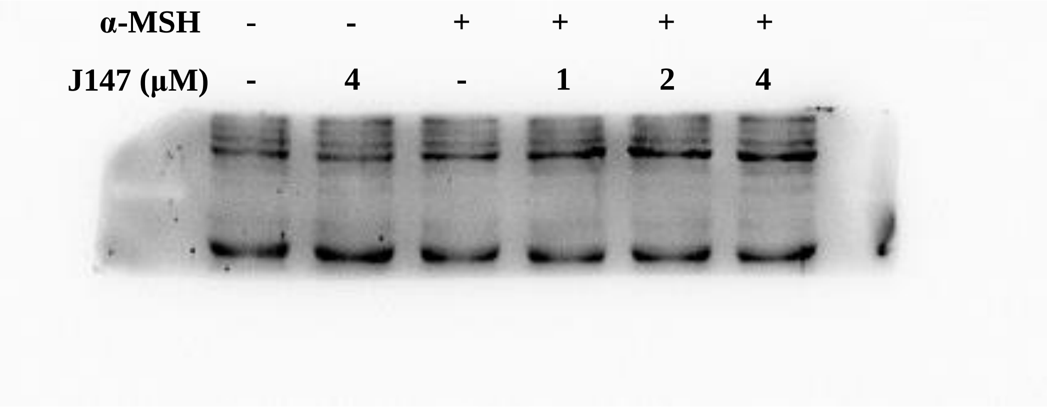

Figure 3B β-actin

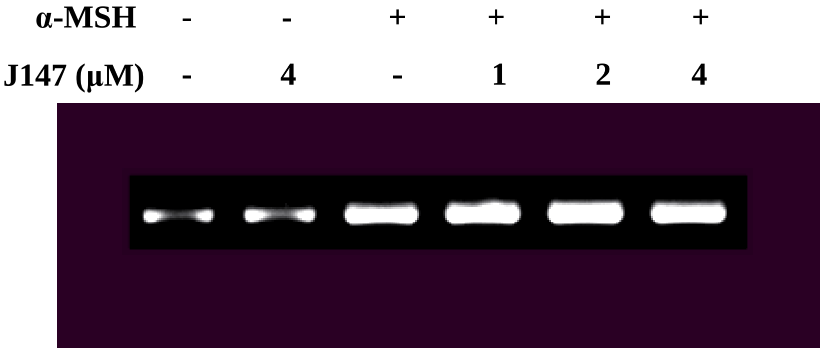

Figure 4A MITF

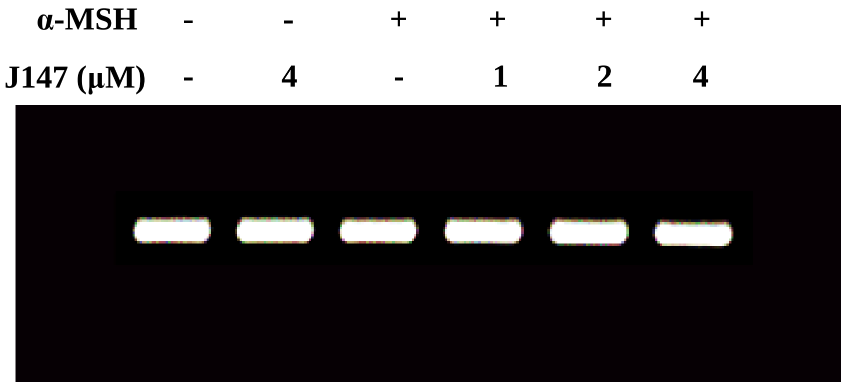

Figure 4A GAPDH

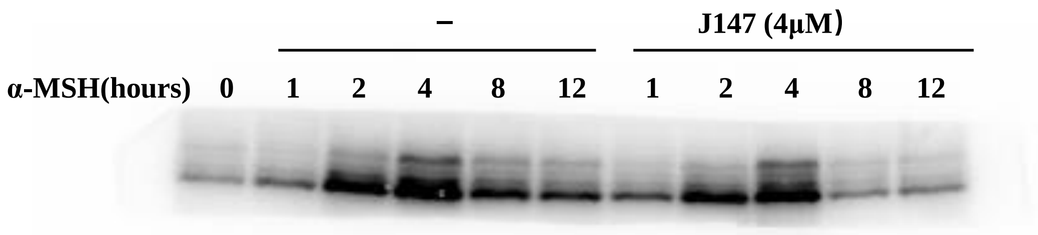

Figure 4B MITF

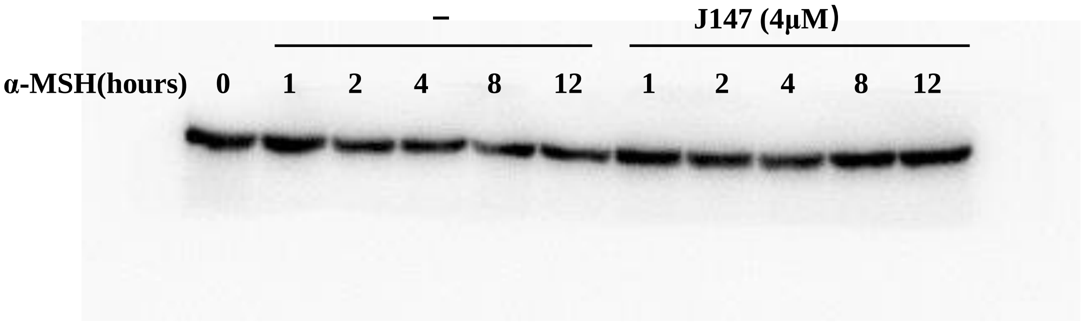

Figure 4B β-actin

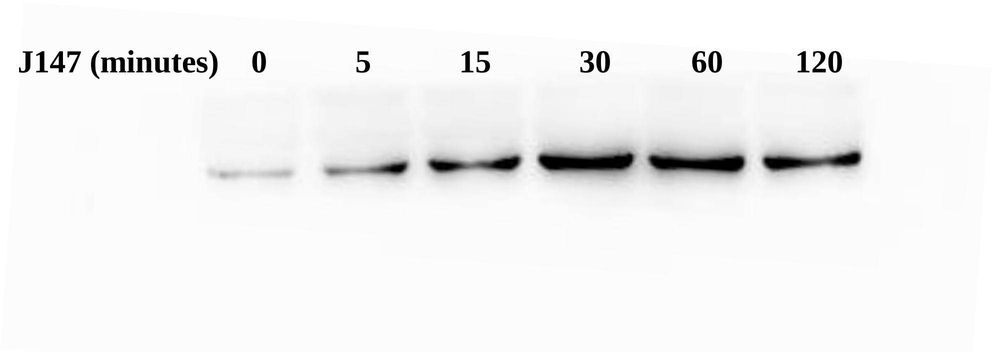

Figure 5A p-MEK

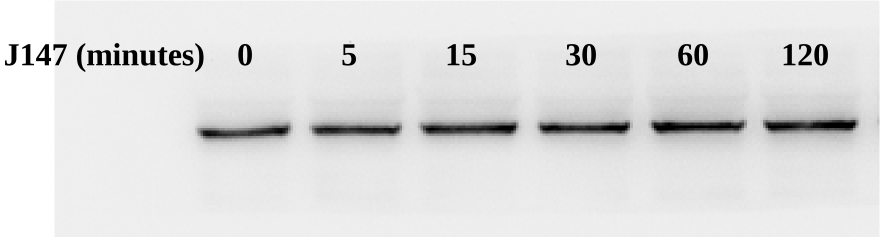

Figure 5A MEK

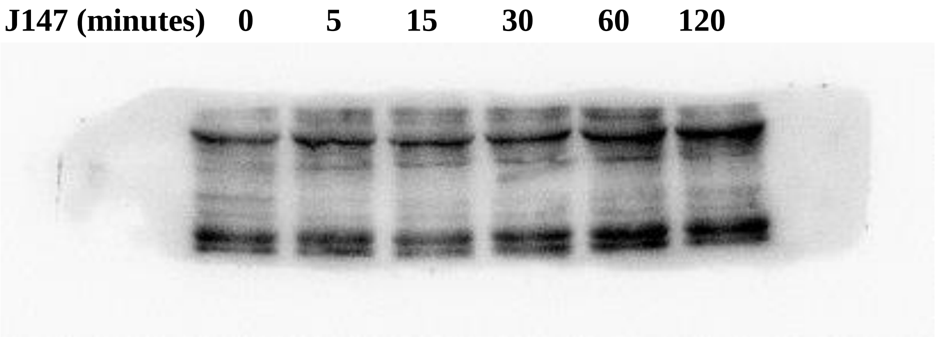

Figure 5A p-ERK

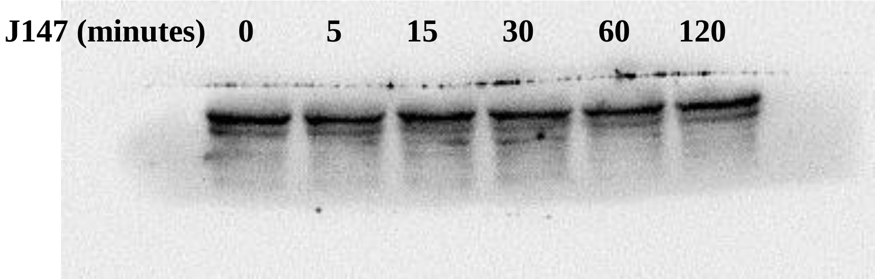

Figure 5A ERK

J147 (minutes) 0 5 15 30 60 120

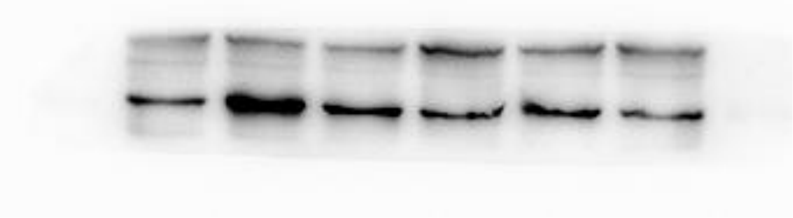

Figure 5A p-p38

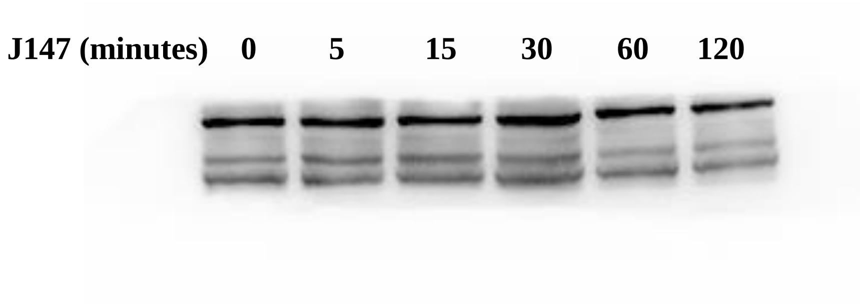

Figure 5A p38

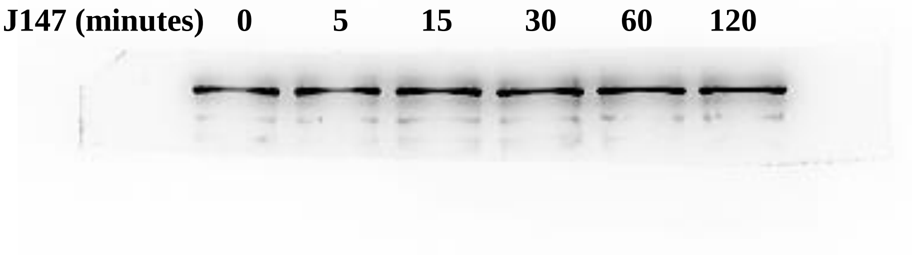

Figure 5A p-JNK

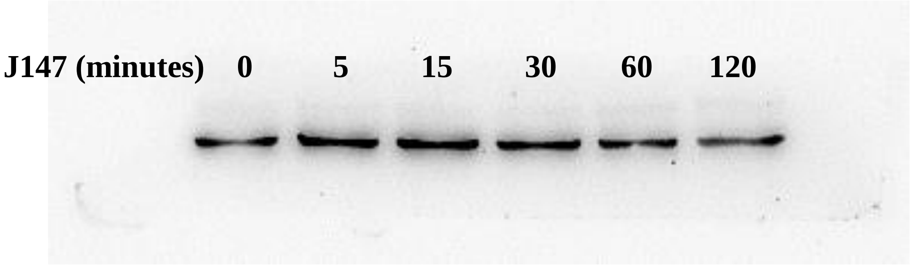

Figure 5A JNK

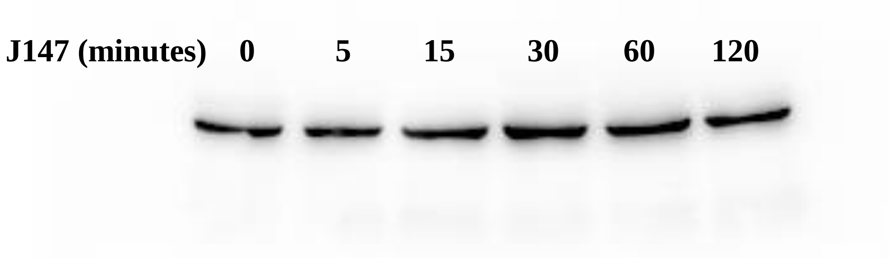

Figure 5A β-actin

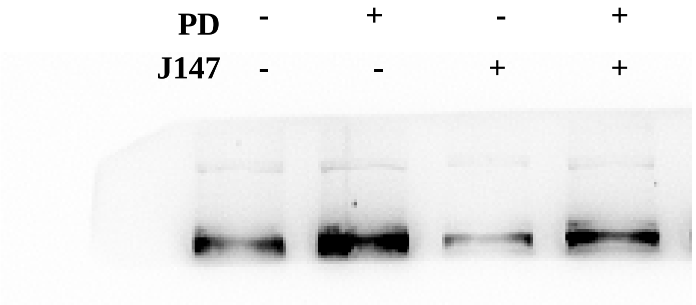

Figure 5B Myosin Va

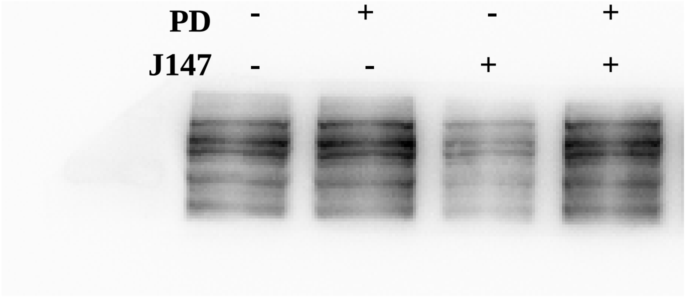

Figure 5B Tyrosinase

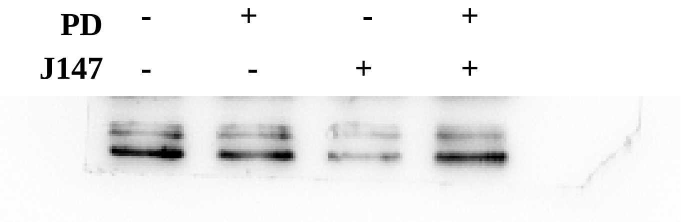

Figure 5B MITF

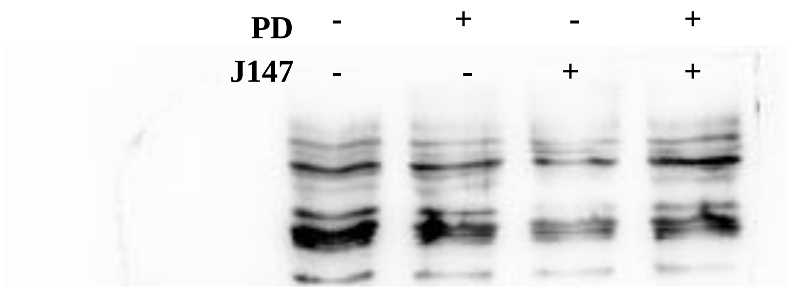

Figure 5B Rab27a

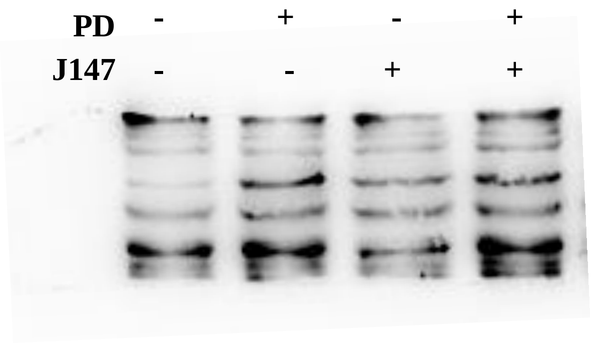

Figure 5B Cdc42

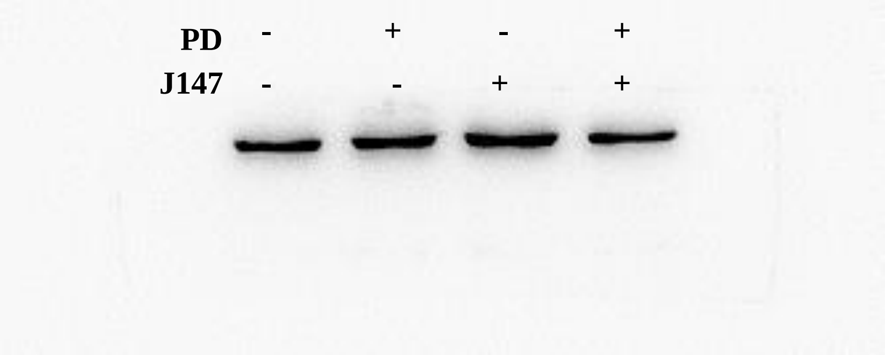

Figure 5B β-actin

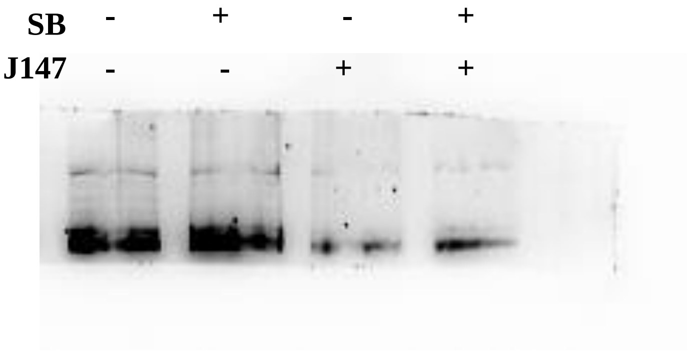

Figure 5C Myosin Va

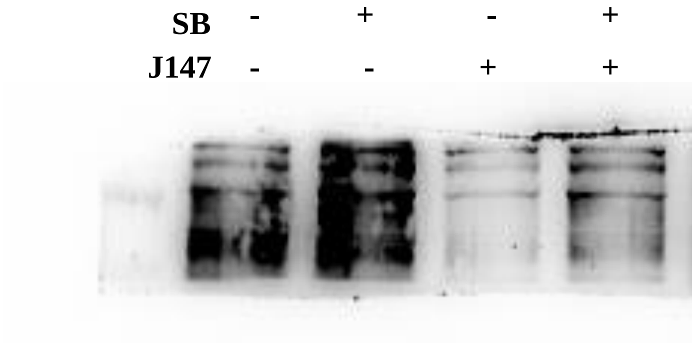

Figure 5C Tyrosinase

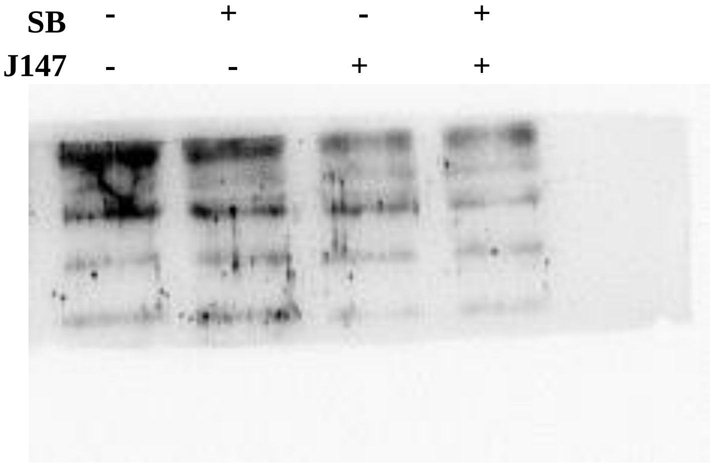

Figure 5C MITF

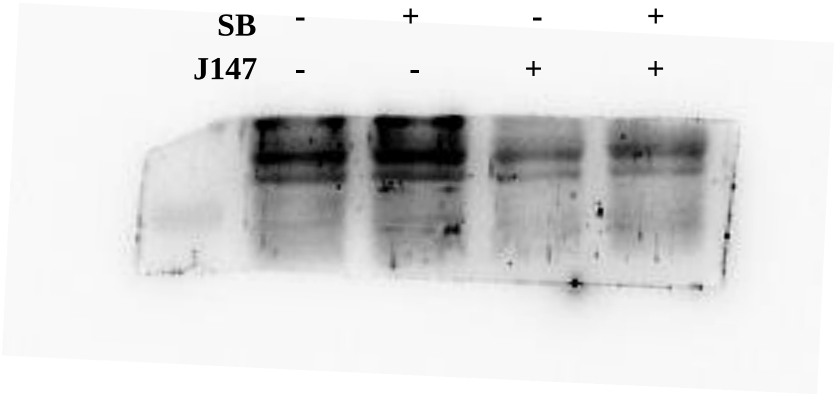

Figure 5C Rab27a

SB	-	+	-	+
J147	-	-	+	+

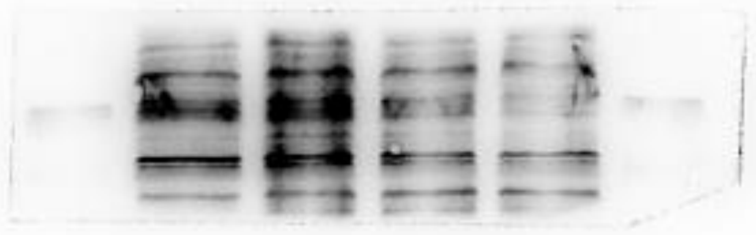

Figure 5C Cdc42

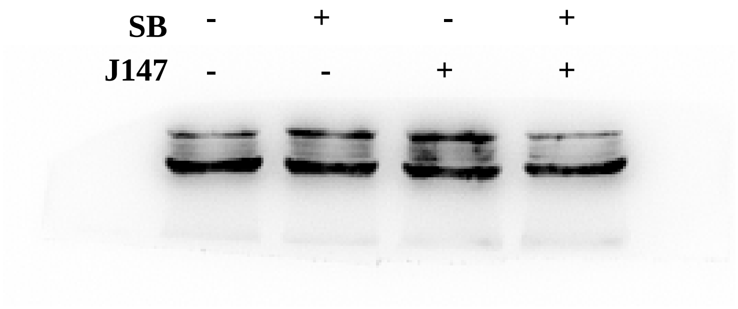

Figure 5C β-actin

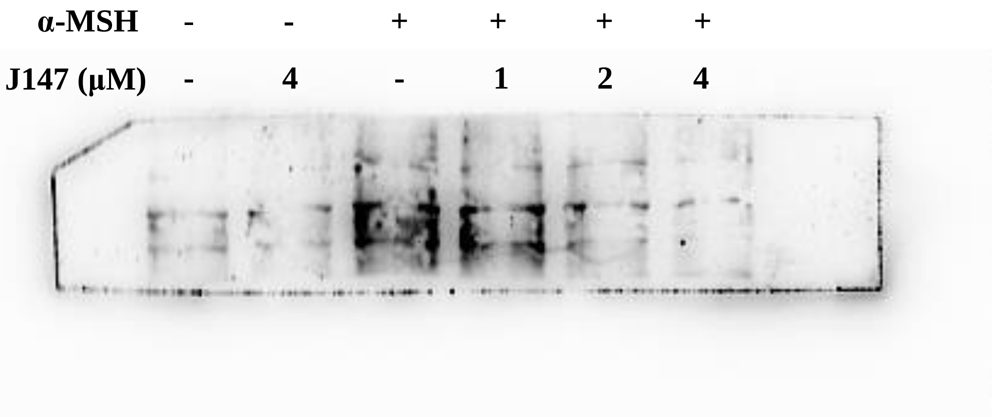

Figure 6B Myosin Va

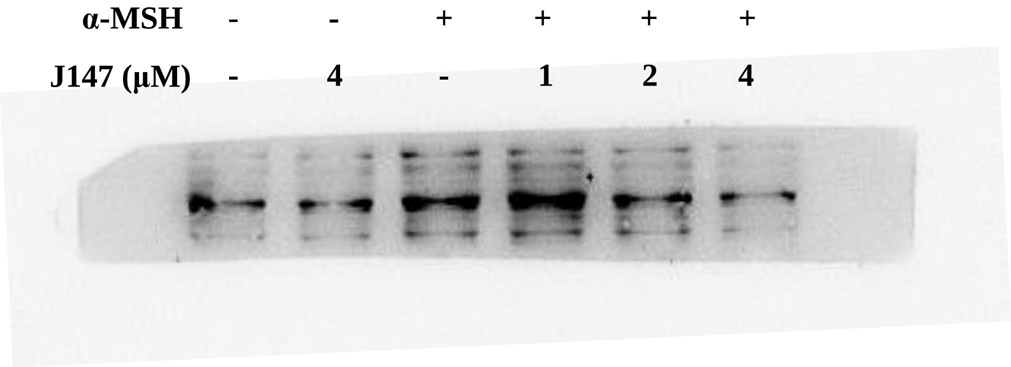

Figure 6B Tyrosinase

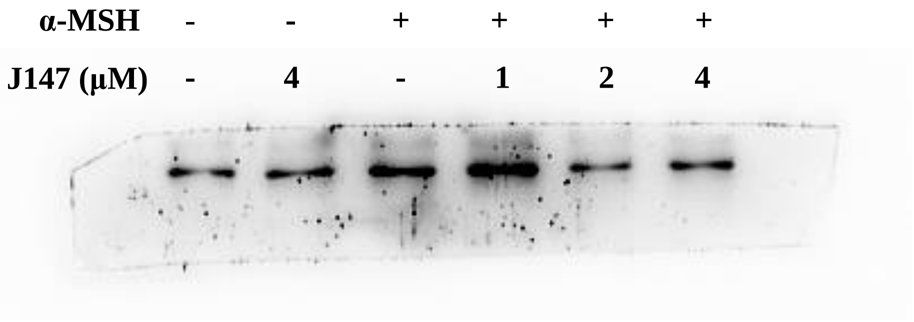

Figure 6B Rab27a

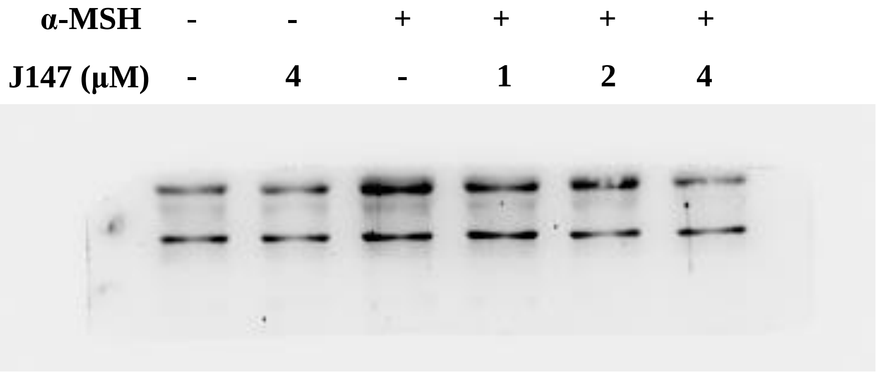

Figure 6B Cdc42

**α-MSH**	-	-	+	+	+	+
**J147 (μM)**	-	4	-	1	2	4

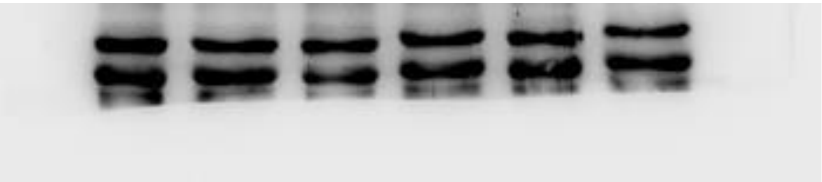

Figure 6B β-actin

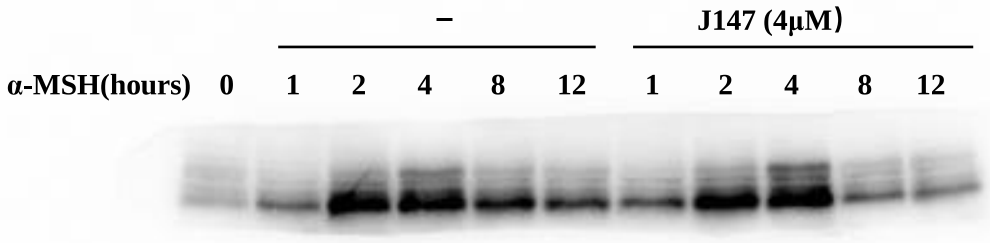

Figure 6C MITF

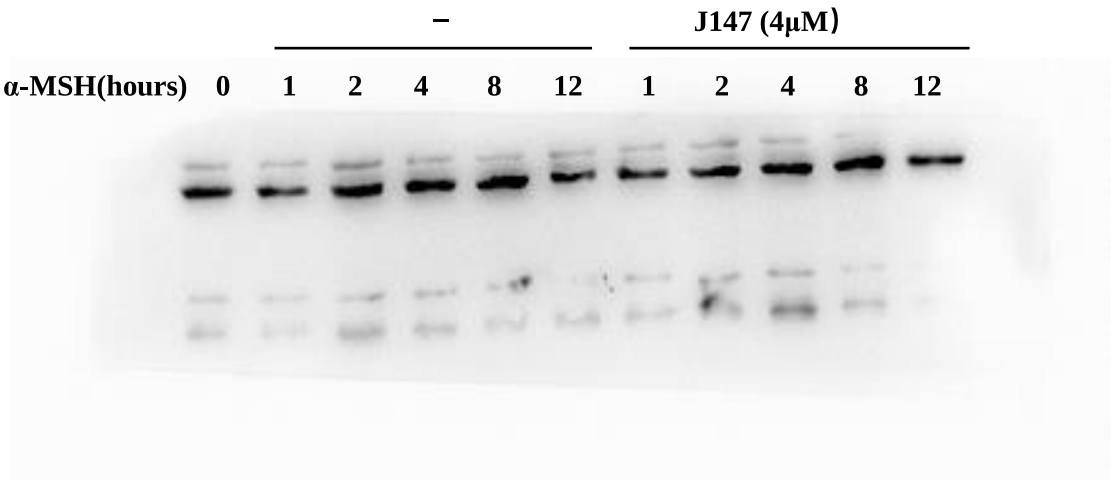

Figure 6C β-actin

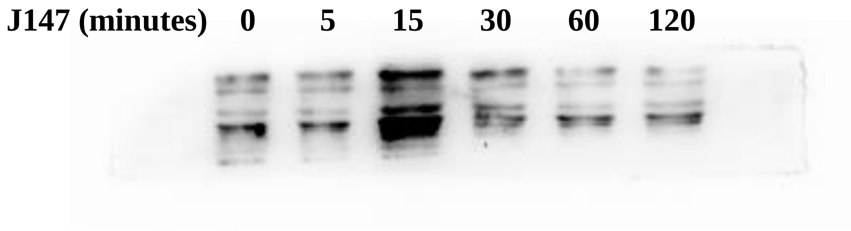

Figure 6D p-MEK

J147 (minutes) 0 5 15 30 60 120

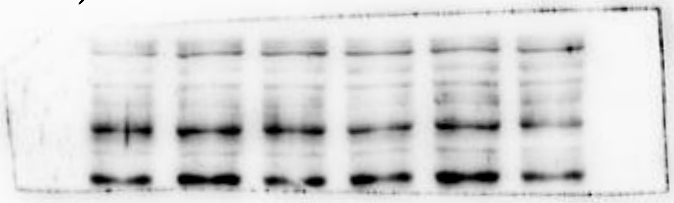

Figure 6D MEK

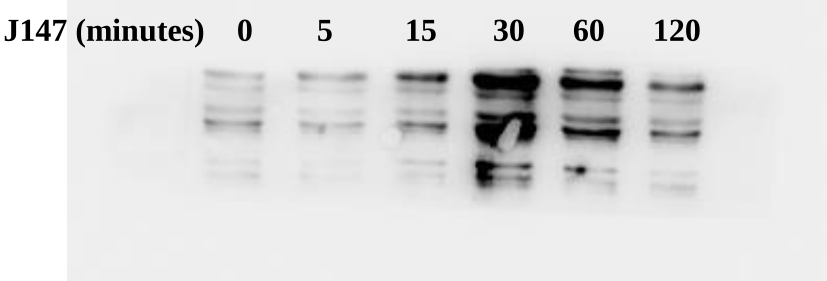

Figure 6D p-ERK

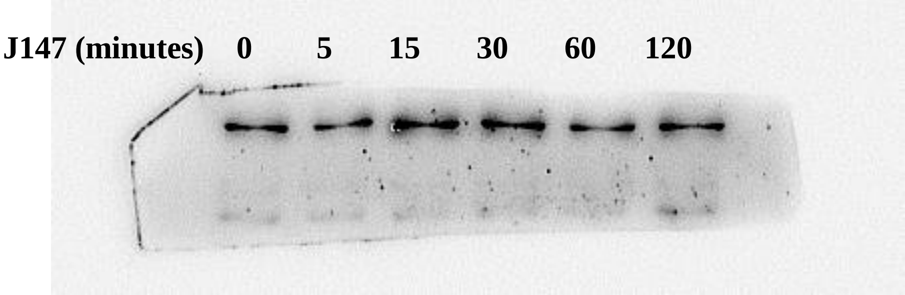

Figure 6D ERK

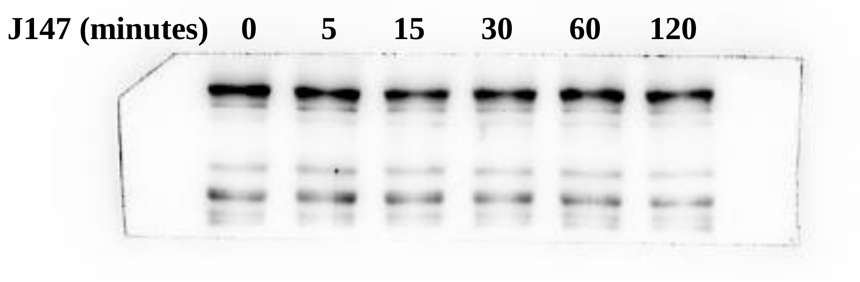

Figure 6D β-actin
