# Supplementary material for: The Inhibitory Effect of Curcumin Derivative J147 on Melanogenesis and Melanosome Transport by Facilitating ERK-Mediated MITF Degradation
Source: Front Pharmacol. 2021 Nov 23;12:783730. doi: 10.3389/fphar.2021.783730 (PMC8649847; doi:10.3389/fphar.2021.783730)
Supplement: Supplementary file 2 [file DataSheet2.DOCX]

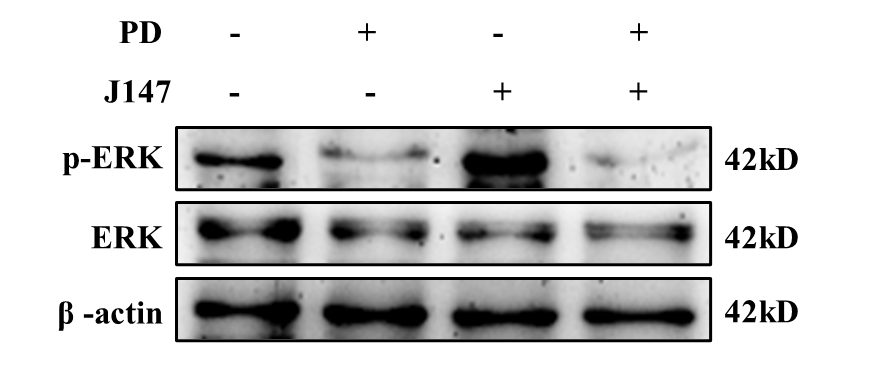


**Figure S1**. Effect of PD98059 on J147-induced ERK activation. B16F10 cells were pretreated with 10 μM PD98059 for 1 hour. Then, B16F10 cells were treated with J147 for 1 hour.


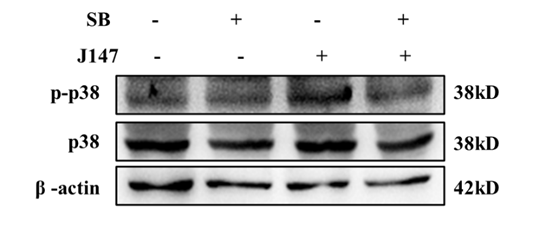


Figure S2. Effect of SB203580 on J147-induced p38 activation. B16F10 cells were pretreated with 10 μM SB203580 for 1 hour. Then, B16F10 cells were treated with J147 for 5 minutes.
